# Aqueous humour cytokines profiles in eyes with Coats disease and the association with the severity of the disease

**DOI:** 10.1186/s12886-020-01421-0

**Published:** 2020-05-05

**Authors:** Tingyi Liang, Yu Xu, Xiuyu Zhu, Xiang Zhang, Jing Li, Peiquan Zhao

**Affiliations:** Department of ophthalmology, Xin Hua Hospital Affiliated to Shanghai Jiao Tong University School of Medicine, Shanghai, 200092 China

**Keywords:** Coats disease, Aqueous humor, Cytokine, Retinal exudation, Exudative retinal detachment

## Abstract

**Background:**

To investigate aqueous humour (AH) cytokine profiles in eyes with Coats disease and analyze the association between cytokine concentrations and the severity of the disease.

**Methods:**

The study included 36 patients (36 eyes) with Coats disease and 15 control patients (15 eyes) with congenital cataract. AH samples were obtained preoperatively and the concentrations of 22 different cytokines were measured through Cytometric Bead Array technology. Clinical characteristics of Coats disease, including the extent of retinal exudation and exudative retinal detachment (ERD), were recorded for analysis.

**Results:**

The concentrations of 8 cytokines (VEGF, IL-6, IL-8, MCP-1, MIP-1α, IP-10, VCAM-1 and ICAM-1) were significantly higher in the Coats disease group than in the control group (all *P* < 0.002). Except for VCAM-1 and ICAM-1, the concentration of the other cytokines listed above showed a significant increase from stage 2 to stage 3 (all *P* < 0.05). Meanwhile, the concentrations of VEGF, IL-8, MCP-1 and MIP-1α showed a significant and positive association with the extent of retinal exudation and ERD (all *r* > 0.4, *P* < 0.05). Among these, IL-8 showed a strong association with the extent of retinal exudation and ERD (all *r* > 0.7, *P* < 0.001). The concentrations of IL-1α, IL-1β, IL-2, IL-4, IL-5, IL-10, IL-12, Fractalkine, RANTES, G-CSF and GM-CSF were very low in both groups.

**Conclusions:**

Various cytokines in the AH, including elevated VEGF, IL-6, IL-8, MCP-1, MIP-1α, IP-10, VCAM-1 and ICAM-1, may be involved in the pathogenesis and progression of Coats disease. Increasing severity of Coats disease is significantly associated with the AH concentrations of VEGF, IL-8, MCP-1 and MIP-1α. Further clinical treatment aimed to reduce vascular leakage and antagonize neovascularization and inflammation may be useful in preventing the progression of Coats disease.

## Background

Coats disease is a rare disorder that predominantly occurs in unilateral eyes of young males. Coats disease is typically characterized by retinal telangiectasia, intraretinal and/or subretinal exudation, and exudative retinal detachment (ERD). In advanced cases, it may progress to total retinal detachment and neovascular glaucoma, which often lead to irreversible visual loss [1].

To date, the etiology of Coats disease remains unclear; however, it is well known that retinal vascular leakage is an important pathological change in Coats disease. Histological findings revealed typical destruction of retinal vascular structure in Coats disease, including the loss of pericytes and endothelial cells, which causes a breakdown of the blood-retinal barrier and increases the vascular permeability, leading to the leakage of a lipid-rich exudate into the retina [2, 3]. Cytokines in the intraocular fluid are indicative of the pathogenesis and progression of ocular diseases [4]. Several previous studies have reported the cytokine changes in eyes with Coats disease [5–8]. Aqueous humour (AH) vascular endothelial growth factor (VEGF) level was highly elevated in eyes with Coats disease and was strongly correlated with the extent of retinal exudation and ERD [5–8]. Meanwhile, anti-VEGF therapy was recommended in clinical treatment of Coats disease, which was reported to be effective in reducing vascular leakage and retinal exudation [9–13]. Aside from VEGF, multiple inflammatory cytokines levels were also investigated. Interleukin (IL)-6, IL-1β and monocyte chemoattractant protein (MCP)-1levels in AH were higher in Coats disease group than the control group, and MCP-1 level was strongly associated with the severity of retinal exudation [7, 8]. Currently, larger sample studies investigating cytokine profiles in Coats disease are needed to further understand the pathogenesis and progression of Coats disease.

In this study, we measured the concentrations of various cytokines in the AH of eyes with Coats disease and analyzed the association between cytokine concentrations and the severity of the disease. We hope to provide new insights into the pathogenesis of Coats disease and lay foundations for further clinical treatment.

## Methods

The present study was conducted at Xinhua Hospital Affiliated to Shanghai Jiao Tong University School of Medicine between June 2016 to June 2018. The study adhered to the tenets of the Declaration of Helsinki and was approved by the Ethics Committee of Xinhua Hospital. Written informed consent was obtained from each patient and their guardians.

### Study subjects

The study enrolled 36 patients (36 eyes) with Coats disease and 15 patients (15 eyes) with congenital cataract as the control group. Coats disease was defined as idiopathic retinal telangiectasia with intraretinal and/or subretinal exudation. The staging of Coats disease was according to classification proposed by Shields et al. [14]. The exclusion criteria of the study group were as follows: (1) the presence of iris neovascularization or anterior chamber cholesterolosis; (2) receiving laser photocoagulation, anti-VEGF therapy or any other treatment previously; (3) a known history of other ocular or systemic disease. Typical clinical characteristics of Coats disease, including the extent of retinal exudation and ERD (clock hours of circumference), were recorded for analysis.

### Sample collection and cytokine assays

The undiluted AH samples (0.1 ml) were obtained before intravitreal anti-VEGF treatment or cataract surgery, and immediately stored at − 80 °C until use. Cytometric Bead Array kit (BD Biosciences, San Diego, CA, USA) was used to measure the concentrations of 22 different cytokines: vascular endothelial growth factor (VEGF), interleukin (IL)-1α, IL-1β, IL-2, IL-4, IL-5, IL-6, IL-8, IL-10, IL-12, monocyte chemoattractant protein (MCP)-1, macrophage inflammatory protein (MIP)-1α, interferon-γ-inducible protein (IP)-10, vascular cell adhesion molecule (VCAM)-1, intercellular cell adhesion molecule (ICAM)-1, basic fibroblast growth factor (bFGF), tumor necrosis factor (TNF)-α, IFN (interferon)-γ, Fractalkine, regulated upon the activation of normal T cell expressed and secreted (RANTES), granulocyte colony-stimulating factor (G-CSF) and granulocyte-macrophage colony-stimulating factor (GM-CSF). Samples were analyzed according to the manufacturer’s instructions. Standard curves for each cytokine were generated using the reference cytokine concentrations supplied in the kit. Cytokine concentration was calculated from a standard curve for each cytokine.

### Statistical analysis

Statistical analysis was performed using Statistical Package for the Social Sciences (SPSS) Version 20 (IBM Corp, Armonk, NY, USA). Data were expressed as the mean ± standard deviation or as median and range. A Shapiro–Wilk test was used to examine whether the variables were distributed normally. Depending on the data distribution, the Student’s *t* test or the Wilcoxon Mann-Whitney test was used to compare the Coats disease group with the control group, and the different stages of Coats disease. Fisher’s exact test was used to compare non-continuous variables. The nonparametric Spearman’s correlation test was used to determine the association between cytokine concentrations and the extent of retinal exudation and ERD. A *P*-value was considered to be statistically significant if *P* < 0.05.

## Results

The demographic information of patients involved in this study was shown in Table 1. The study group included 36 eyes of 36 patients (31 boys) with Coats disease, and the control group consisted of 15 eyes of 15 patients (4 boys) with congenital cataract. The average age of Coats disease and control groups were 4.3 years and 3.1 years, respectively. In Coats disease group, the eye was classified as stage 2 in 15 cases (41.7%) and stage 3 in 21 cases (58.3%).

**Table 1:** Characteristics of patients and eyes in the Coats disease group and the control group

	Coats disease	Congenital cataract	*P*-Value
No. of patients/eyes	36 patients/36 eyes	15 patients/15 eyes	–
Mean age ± SD, years	4.3 ± 2.4	3.1 ± 1.1	0.070^#^
No. of male patients (%)	31 (86.1%)	11 (73.3%)	0.285^*^
No. of eyes at different stages (%)			
stage2	15 (41.7%)	–	–
stage3	21 (58.3%)	–	–

*SD* standard deviation

# Student’s *t* test

* Fisher’s exact test

**Fig. 1:**
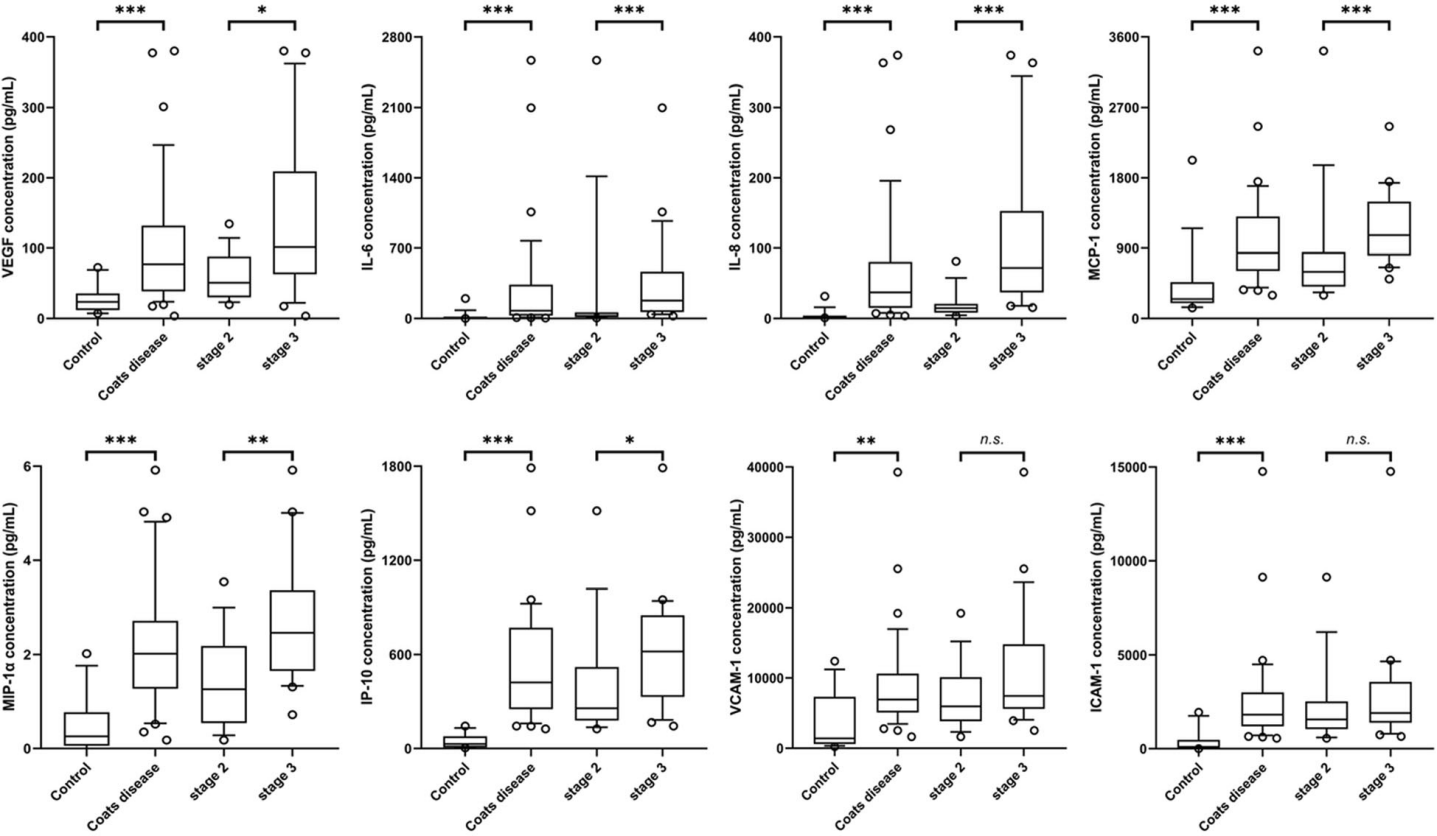
Aqueous humour cytokine concentrations of VEGF, IL-6, IL-8, MCP-1, MIP-1α, IP-10, VCAM-1 and ICAM-1 in the control, Coats disease, stage 2 and stage 3 groups (the median and 25th to 75th percentiles: boxes, 10th to 90th percentiles: whiskers, outliners: circles). *n.s.* not significant; ***P < 0.001, **P < 0.01, *P < 0.05

The results of AH cytokine analysis were shown in Table 2. The limit of detection (LOD) for each cytokine were included. Of the 22 measured cytokines, the concentrations of 8 cytokines (VEGF, IL-6, IL-8, MCP-1, MIP-1α, IP-10, VCAM-1 and ICAM-1) were significantly higher in the Coats disease group than in the control group (all *P* < 0.002). Of the 8 cytokines, the concentrations of VEGF, IL-6, IL-8, MCP-1, MIP-1α and IP-10 showed a significant increase from stage 2 to stage 3 (all *P* < 0.05) (Fig. 1). No significant difference was observed in bFGF, TNF-α and IFN-γ between the Coats disease group and the control group. The rest of cytokines, including IL-1α, IL-1β, IL-2, IL-4, IL-5, IL-10, IL-12, Fractalkine, RANTES, G-CSF and GM-CSF, were at very low concentration and close to LOD in both groups.

**Table 2:** Aqueous humour cytokine concentrations (pg/mL) in the Coats disease group and the control group

Cytokines	Coats disease (*n* = 36)	stage 2 (*n* = 15)	stage 3 (*n* = 21)	Control Group (*n* = 15)	*P*-Value^#^	*P*-Value^*^	LOD
VEGF	77.1 (3.4–379.9)	50.9 (19.7–134.4)	101.2 (3.4–379.9)	23.6 (7.2–72.7)	**< 0.001**	**0.013**	4.5
IL-1α	0 (0–1.8)	0 (0–0)	0 (0–1.8)	0 (0–1)	1.0	0.063	1.0
IL-1β	0.8 (0–3.9)	0.7 (0–1.4)	1 (0.3–3.9)	0.7 (0–3.9)	0.329	0.016	2.3
IL-2	4.6 (0–15.5)	2.9 (0.3–8.2)	4.9 (0–15.5)	3.9 (0.6–10.5)	0.756	0.080	11.2
IL-4	1.7 (0.7–2.3)	1.7 (1.2–2.1)	1.6 (0.7–2.3)	1.1 (0.6–1.7)	< 0.001	0.168	1.4
IL-5	1.1 (0–21.3)	0.6 (0–1.3)	2.3 (0–21.3)	0.6 (0–1.3)	0.448	0.001	1.1
IL-6	77.0 (5.3–2566.1)	26.8 (5.3–2566.1)	179.2 (24.9–2096.4)	0.6 (0–199.2)	**< 0.001**	**< 0.001**	1.6
IL-8	37.2 (3.7–373.8)	14.7 (3.7–81.5)	71.9 (15.4–373.8)	2 (0.9–31.6)	**< 0.001**	**< 0.001**	1.2
IL-10	0.1 (0–3.2)	0 (0–0.4)	0.2 (0–3.2)	0.4 (0–3)	0.035	0.002	0.1
IL-12	0.2 (0–22.7)	0.2 (0–1.8)	0.1 (0–22.7)	0 (0–0.3)	0.016	0.194	0.6
MCP-1	837.1 (298.5–3420)	596.6 (298.5–3420)	1065.2 (505.8–2458.1)	248.4 (135.3–2024.7)	**< 0.001**	**< 0.001**	1.3
MIP-1α	2 (0.1–5.9)	1.2 (0.1–3.5)	2.4 (0.7–5.9)	0.2 (0–2)	**< 0.001**	**0.003**	0.2
IP-10	421.2 (125.2–1790.2)	257 (125.2–1515.4)	618.8 (143.2–1790.2)	29.5 (3.8–143)	**< 0.001**	**0.010**	0.5
VCAM-1	6925.4 (1644.3–39,264.4)	5950.6 (1644.3–19,217.3)	7440.3 (2530.1–39,264.4)	1407.1 (230.9–12,390.3)	**0.001**	0.205	12.2
ICAM-1	1816.2 (559.5–14,769.5)	1557 (559.5–9125)	1903.6 (654.1–14,769.5)	100.9 (2.7–1938.8)	**< 0.001**	0.221	25.7
bFGF	10.5 (1–117.4)	8.6 (1–117.4)	11.7 (1.9–28.8)	13.5 (3.3–29.4)	0.162	0.130	3.4
TNF-α	3.7 (0–74.4)	2.8 (0–74.4)	4 (0–9.7)	3.2 (0.4–6.4)	0.882	0.189	1.2
IFN-γ	2.9 (0.2–9.4)	2.4 (0.2–3.6)	3.3 (1.4–9.4)	2.8 (0.1–5.9)	0.539	0.011	0.8
Fractalkine	3.8 (0–23.9)	6.3 (1.5–23.9)	2.7 (0–10.8)	4.2 (0–23.9)	0.680	0.003	22.3
RANTES	0.5 (0.1–20.4)	0.5 (0.4–2.3)	0.6 (0.1–20.4)	0.4 (0–64.2)	0.003	0.046	0.0
G-CSF	1.8 (1–4.7)	1.7 (1–2.7)	1.9 (1.1–4.7)	1.1 (0.6–1.4)	< 0.001	0.040	1.6
GM-CSF	0.9 (0–3.8)	0.9 (0–1.8)	0.9 (0.4–3.8)	1.0 (0–3.9)	0.687	0.254	0.2

Values in bold font are statistically significant at *P* < 0.05

*LOD* limit of detection, *VEGF* vascular endothelial growth factor, *IL* interleukin, *MCP-1* monocyte chemoattractant protein-1, *MIP-1α* macrophage inflammatory protein-1α, *IP-10* interferon-γ-inducible protein-10, *VCAM* vascular cell adhesion molecule, *ICAM* intercellular cell adhesion molecule, *bFGF* basic fibroblast growth factor, *TNF-α* tumor necrosis factor-α, *IFN-γ* interferon-γ, *RANTES* regulated upon the activation of normal T cell expressed and secreted, *G-CSF* granulocyte colony-stimulating factor, *GM-CSF* granulocyte macrophage colony-stimulating factor

^#^Wilcoxon Mann–Whitney test was used for comparisons between the Coats disease group and the control group

^*^Wilcoxon Mann–Whitney test was used for comparisons between stage 2 and stage 3

**Fig. 2:**
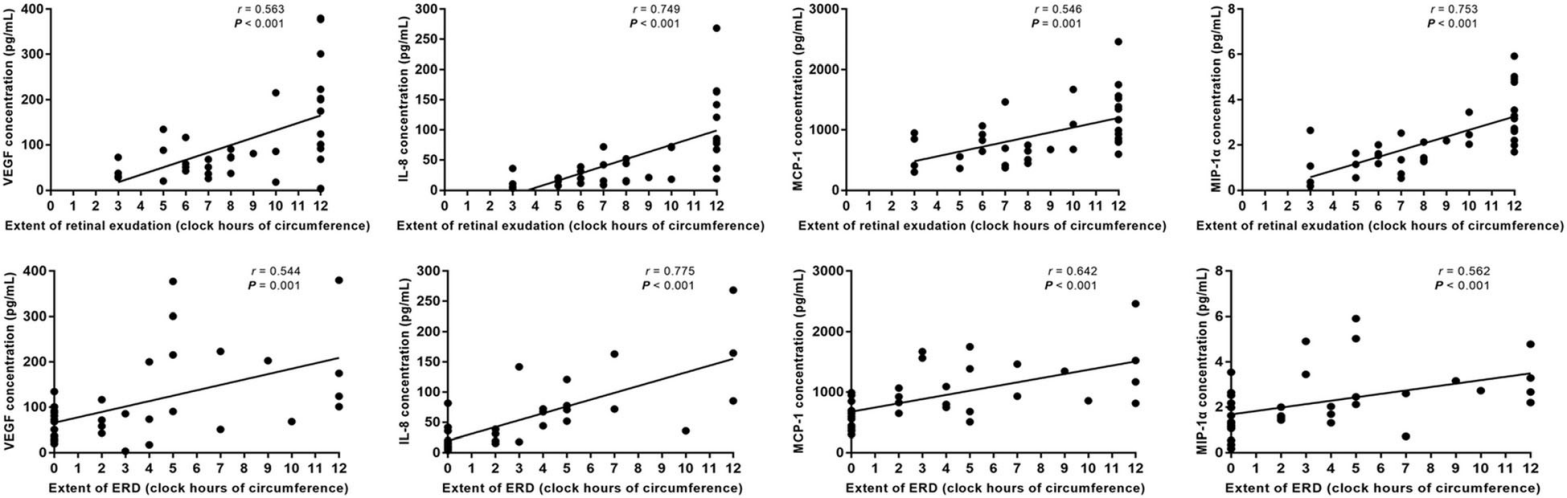
The association of aqueous humour cytokine concentrations of VEGF, IL-8, MCP-1 and MIP-1α with the extent of retinal exudation and exudative retinal detachment (ERD) in eyes with Coats disease. The number of *X*-axis represents the number of circumferential clock hours (1–12) displaying retinal exudation or ERD

We further analyzed the association of cytokine concentrations of VEGF, IL-6, IL-8, MCP-1, MIP-1α, IP-10, VCAM-1 and ICAM-1 with the extent of retinal exudation and ERD. The results were shown in Table 3. We found a significant and positive association between the concentrations of VEGF (*r* = 0.563, *P* < 0.001), IL-8 (*r* = 0.749, *P* < 0.001), MCP-1 (*r* = 0.546, *P* = 0.001), MIP-1α (*r* = 0.753, *P* < 0.001), IP-10 (*r* = 0.417, *P* = 0.011) and ICAM-1 (*r* = 0.401, *P* = 0.019) and the extent of retinal exudation. The extent of ERD was significantly and positively associated with concentrations of VEGF (*r* = 0.544, *P* = 0.001), IL-6 (*r* = 0.607, *P* < 0.001), IL-8 (*r* = 0.775, *P* < 0.001), MCP-1 (*r* = 0.642, *P* < 0.001) and MIP-1α (*r* = 0.562, *P* < 0.001). Among these, IL-8 showed a strong association with the extent of retinal exudation and ERD (all *r* > 0.7, *P* < 0.001). The concentration of VCAM-1 was not significantly associated with either retinal exudation or ERD. Representative scatter plots of the association of AH cytokine concentrations with the extent of retinal exudation and ERD were shown in Fig. 2.

**Table 3:** Association of aqueous humour cytokine concentrations with the extent of retinal exudation and exudative retinal detachment

Cytokines	Retinal exudation		Exudative retinal detachment	
	*r*	*P*-Value^#^	*r*	*P*-Value^#^
VEGF	0.563	**< 0.001**	0.544	**0.001**
IL-6	0.338	0.051	0.607	**< 0.001**
IL-8	0.749	**< 0.001**	0.775	**< 0.001**
MCP-1	0.546	**0.001**	0.642	**< 0.001**
MIP-1α	0.753	**< 0.001**	0.562	**< 0.001**
IP-10	0.417	**0.011**	0.328	0.051
VCAM-1	0.284	0.099	0.280	0.103
ICAM-1	0.401	**0.019**	0.309	0.076

Values in bold font are statistically significant at *P* < 0.05

*VEGF* vascular endothelial growth factor, *IL* interleukin, *MCP-1* monocyte chemoattractant protein-1, *MIP-1α* macrophage inflammatory protein-1α, *IP-10* interferon-γ-inducible protein-10, *VCAM* vascular cell adhesion molecule, *ICAM* intercellular cell adhesion molecule

^#^ Spearman’s correlation test was used to determine the association of cytokine concentrations with the extent of retinal exudation and exudative retinal detachment

## Discussion

In the present study, we reported AH cytokine profiles in eyes with Coats disease in a relatively large sample size. We found that the concentrations of 8 out of 22 cytokines (VEGF, IL-6, IL-8, MCP-1, MIP-1α, IP-10, VACM-1 and ICAM-1) were significantly increased in the AH of eyes with Coats disease. Among which, the concentrations of VEGF, IL-8, MCP-1, MIP-1α were significantly associated with the extent of retinal exudation and ERD. The above elevated cytokines are involved in angiogenesis, increased vascular permeability and inflammatory response in the retina. The present study broadens the understanding of the pathogenesis of Coats disease, which may be valuable for further clinical treatment.

Consistent with previous studies [6–8], we found significantly increased VEGF concentration in the AH of eyes with Coats disease; furthermore, the VEGF concentration was positively associated with the increasing severity of the retinal exudation and ERD. VEGF is an important proangiogenic cytokine and is also associated with vascular leakage in the retina [15]. An immunohistopathological study reported that VEGF was highly expressed by infiltrated macrophage in enucleated eyes with Coats disease, which contained typical retinal vascular abnormalities [16]. Clinically, mounting studies confirmed the effect of intravitreal anti-VEGF therapy by reducing vascular leakage and retinal exudation in Coats disease [5, 9–11]. Therefore, VEGF may act an important role in the general pathogenesis of Coats disease and may be one of driving force in stimulating vascular leakage and neovascularization. Additionally, the present study showed a significant increase of VEGF concentration from stage 2 to stage 3, which implies that anti-VEGF therapy in the early stage of Coats disease appears to be effective in preventing disease progression.

Previous studies suggested that inflammation might be involved in the pathogenesis of Coats disease [7, 8], and intravitreal anti-inflammation therapy was reported to have a certain treatment effect on Coats disease [17–19]. However, the molecular mechanisms are still poor understood. Coats disease may be not a classic inflammatory disease, which is supported by AH cytokine profiles revealed in this study. Despite the increase of IL-6, IL-8, MCP-1 and MIP-1α, we found no comparable increase of IL-1β or TNF-α, which were hallmarks of inflammatory activation of macrophage [20]. In addition, we also found no increase of typical cytokines associated with T- or B-lymphocyte activation, such as IL-2, IL-4, IL-5, IL-10, IL-12 and IFN-γ [21–24]. Thus, inflammatory activation of macrophage, and T- and B-lymphocyte mediated inflammatory responses may be limited in Coats disease.

Although inflammation may be not a prominent feature in Coats disease, inflammatory cells such as macrophage and T-lymphocyte were identified in enucleated eyes with Coats disease [16, 25]. The pathological change of retinal vascular structure causes the destruction of blood-retinal barrier, which makes it possible to collect inflammatory cells at the perivascular spaces and leads to higher concentrations of intraocular inflammatory cytokines. Correspondingly, changes in inflammatory cytokines suggested a possible association between the aggravation of the disease and the intensification of inflammation. In our study, the concentrations of inflammatory cytokines, including IL-6, IL-8, MCP-1 and MIP-1α, were significantly increased, and showed a progressive increase in parallel with the disease stage; moreover, the concentrations of IL-8, MCP-1 and MIP-1α showed moderate to strong associations with the severity of retinal exudation and ERD. The above elevated inflammatory cytokines are important proinflammatory factors, and also involve the regulation of angiogenesis and the increase of vascular permeability [26–28]. These inflammatory cytokines may participate the progression of Coats disease; however, their sources and true roles in Coats disease remain to be investigated. Based on the cytokine profiles in eyes with Coats disease, further studies designing to probe the identity of different cellular components and their state of activation may provide important information to unveil the mechanisms driving disease progression.

Other significantly elevated cytokines in our study should be noted. VCAM-1 and ICAM-1 play important roles in traversing leukocytes across endothelial cells, which also exacerbate the destruction of blood-retinal barrier [29, 30]. In the present study, significantly elevated VCAM-1 and ICAM-1 provide a possible molecular evidence for increased vascular permeability and accumulation of inflammatory cells in Coats disease. However, the above two cytokines showed no increase from stage 2 to stage 3, suggesting that vascular permeability may not change during the disease progression. IP-10, a potent T-lymphocyte chemoattractant [31], was also found significantly elevated, which may contribute to T-lymphocyte attraction in Coats disease.

The present study has several limitations. Firstly, compared with AH samples used in our study, the cytokine profiles of vitreous and/or subretinal fluid samples may better reflect fundus condition, however, which is limited by the need for more invasive approaches. Secondly, retinal fibrosis, one of the common clinical manifestations in Coats disease, and its association with AH cytokine concentrations should be taken into account. However, retinal fibrosis is usually late-onset in Coats disease [32]; in our study, none of the patients showed retinal fibrosis at presentation. Further studies investigating the association of AH cytokines with retinal fibrosis in Coats disease are needed. Last but not least, there may be some cytokines associated with Coats disease that the present study has not fully covered. In further studies, using a protein array chip to detect abundant proteins in the AH may be more helpful for a comprehensive understanding of the pathogenesis of Coats disease.

## Conclusions

In conclusion, retinal vascular leakage is the fundamental pathological change in Coats disease, accompanied by the increase of various AH cytokines, including VEGF, IL-6, IL-8, MCP-1, MIP-1α, IP-10, VACM-1 and ICAM-1, which is involved in angiogenesis, increased vascular permeability and inflammatory response and may contribute to the pathogenesis and progression of the disease. Increasing severity of Coats disease is significantly associated with AH concentrations of VEGF, IL-8, MCP-1 and MIP-1α. In the future, treatment aimed to reduce vascular leakage and antagonize neovascularization and inflammation may be useful in preventing the progression of Coats disease.

## Data Availability

The datasets used and analyzed during the current study are available from the corresponding author on reasonable request.

## Abbreviations

ERD: Exudative retinal detachment
AH: Aqueous humour
VEGF: Vascular endothelial growth factor
IL: Interleukin
MCP-1: Monocyte chemoattractant protein-1
MIP-1α: Macrophage inflammatory protein-1α
IP-10: Interferon-γ-inducible protein-10
VACM-1: Vascular cell adhesion molecule-1
ICAM-1: Intercellular cell adhesion molecule-1
bFGF: Basic fibroblast growth factor
TNF-α: Tumor necrosis factor-α
IFN-γ: Interferon-γ
RANTES: Regulated upon the activation of normal T cell expressed and secreted
G-CSF: Granulocyte colony-stimulating factor
GM-CSF: Granulocyte macrophage colony-stimulating factor
